# Soil Nutrient Availability Drives Fungal Community Structure and Function During the Transformation of Eucalypt Plantations Logging Sites in Southern China

**DOI:** 10.1002/ece3.72263

**Published:** 2025-11-12

**Authors:** Yuxing Xu, Zhichao Wang, Runxia Huang, Wankuan Zhu, Apeng Du, Chao Li

**Affiliations:** ^1^ Chinese Academy of Forestry (CAF) Research Institute of Fast‐Growing Trees (RIFT) Zhanjiang China

**Keywords:** co‐occurrence network, fungal community, FUNGuild, Illumina MiSeq sequencing, soil physico‐chemical properties

## Abstract

Highly intensive eucalypt plantation management has led to nutrient imbalances in forest land, severely weakening the ecosystem's self‐healing ability. Thus, restoring logging sites is urgent. Soil microorganisms, particularly fungi, are crucial in soil ecosystems for promoting nutrient cycling, regulating soil properties, and maintaining balance. Yet, how degraded logging site transformation patterns affect the structure and function of subsurface ecosystem components remains unclear. This study examined the impacts of four transformation patterns on soil physicochemical properties and fungal communities in eucalypt logging sites and their relationships. The soil dissolved organic carbon, nitrate nitrogen (${\mathrm{NO}}_{3}^{-}$_N), and available phosphorus (AP) contents in the mixed plantations of *Eucalyptus* × *Manglietia glauca* (EM) and the rotational plantations of *M. glauca* (MM) were higher than those in the slope unforested grasses and shrubs lands (GS) and the continuous‐cropping eucalypt plantations (EE). Correspondingly, both EM and MM increased the relative abundances (RAs) of Mortierellomycetes and Rozellomycotina_cls_Incertae_sedis, which have a broad range of cellulose‐ and lignin‐degrading capabilities. In contrast, EE significantly increased the dependence of eucalypt on ectomycorrhizal fungi (mostly belonging to Agaricomycetes) and reduced soil fungal network complexity and stability compared to other transformation patterns. EM and MM significantly increased saprophytic fungi RAs and showed higher fungal network complexity compared with the above two patterns. These two patterns also distinctly inhibited the proportion of soil pathogenic fungi and the soil acidification process. Mantel test and redundancy analysis showed that fungal community composition and function were jointly driven by pH, ${\mathrm{NO}}_{3}^{-}$_N, and AP contents. Additionally, during the dry season, soil water content significantly influenced fungal community composition and function. These findings have important implications for restoring degraded logging sites in eucalypt plantations.

## Graphical abstract

Highly intensive eucalyptus plantation management has led to an imbalance of nutrients in the forest land, which has seriously weakened the self‐healing capacity of the ecosystem. Therefore, it is urgent to restore the soil quality of logging sites in multi‐generation continuous plantations. Soil microorganisms, especially fungi, play an important role in soil ecosystems to promote nutrient cycling, regulate soil properties and maintain balance. Based on this, this experiment investigated the effects of four transformation patterns on soil physicochemical properties and fungal communities in eucalyptus logging sites and their interrelationships.

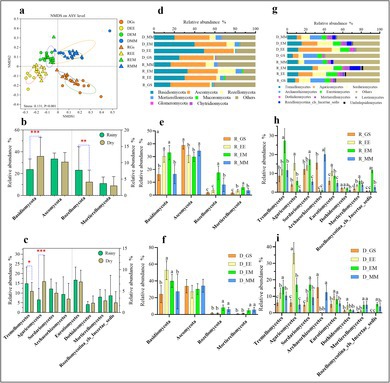

## 1 Introduction

In recent times, due to the increasing global population and demand for forest‐based products, the decline process of the primary forest area has gradually accelerated (Kirilenko and Sedjo 2007). Thus, vigorously developing plantations have become important worldwide management practices for restoring forest growth and counteracting forest depletion (Evans 2009; Teixeira et al. 2017). Eucalypt has been selected as an important national strategic tree species in many areas of southern China due to its resistance to soil infertility and high wood yield (Chen et al. 2011). However, approximately 80% of the eucalypt plantation area adopts the short rotation and continuous planting pattern, which has caused many ecological and environmental problems, such as ecosystem stability decline and biodiversity degradation due to highly intensive management (Xu et al. 2020; Xu, Li, et al. 2021; Wang et al. 2022; Bose et al. 2023). Many large‐scale state‐owned forest farms have adopted management measures of building eucalypt mixed plantations with native tree species to gradually restore degraded forest ecosystems and compensate for the negative impacts of degraded plantation ecosystems. Consequently, these practices ensure appropriate management benefits, achieving favorable pattern results in terms of soil fertility and carbon (C) stock enhancement (Huang et al. 2017; Xu et al. 2022; Zhang, You, et al. 2023). However, feedback on the effects of degraded forest management practices on the structure and function of belowground ecosystem components has received little attention, limiting the overall evaluation of restoration efforts in degraded ecosystems.

Soil microorganisms, as the most abundant organic organisms in subsurface ecosystems, are important drivers of soil nutrient cycling, physical property improvement, and organic matter degradation (Guerra et al. 2020). Soil microorganisms, as the most abundant organic organisms in the subsurface ecosystem, are important drivers of soil nutrient cycling and organic matter degradation. Changes in soil microbial community composition and diversity are often used as important indicators for evaluating forest productivity and soil health (Xu, Li, et al. 2021; Selvalakshmi et al. 2022). For example, Xu, Li, et al. (2021) found that highly intensive management of eucalypt caused significant declines in soil functional diversity, microbial abundance, and co‐occurrence network stability, especially after the third generation of continuous planting. Similarly, negative cases of soil fertility degradation decreased microbial metabolic activity, and the accumulation of pathogenic fungi caused by irrational management patterns was found in both Chinese fir and pine plantations (Wu et al. 2015, 2017). Although the adverse effects of irrational management on soil health have been widely studied, research on the restoration patterns of degraded woodland harvesting sites is relatively scarce. In particular, changes in the soil microbial community structure and the associated biochemical cycling processes induced by the restoration process remain unclear.

Fungi, as integral components of soil microorganisms, affect forest productivity by participating in different parts of the material cycle and the energy flow of ecosystems (Castano et al. 2019). In particular, fungi, as decomposers, regulate the soil C balance and drive nutrient cycling by secreting a variety of extracellular enzymes (Treseder and Lennon 2015; Žifčáková et al. 2016). Additionally, fungi, such as soil pathogens, are able to regulate microbial species composition and community structure (Van Agtmaal et al. 2017). At the same time, symbiotic fungi are able to maintain mutually beneficial relationships with their hosts (Li, Xu, et al. 2023). Studies have confirmed that the number of fungi surviving as saprophytic or symbiotic forms in acidic forest soils in subtropical regions exceeds the number of bacteria involved in decomposition (Maraun and Scheu 1996). It has also been shown that fungal communities may play a more important role in above‐ and belowground connectivity and C, N, and P cycling than bacterial communities (Jin et al. 2019). Given the importance of soil fungi in ecosystems, understanding the impact of degraded plantation restoration practices on soil fungal communities is of practical significance for developing rational agroforestry ecosystem management measures (Gao et al. 2022).

Accordingly, this study set up four transformation patterns based on logging sites that had undergone two generations of *Eucalyptus urophylla* × *E. grandis* planting: (1) suppression of logging stump sprouting to form an unforested area dominated by grasses and shrub lands (GS), (2) continuously cultivating third‐generation pure eucalypt plantations (EE), (3) building eucalypt and *M. angustifolia glauca* mixed plantations (EM), and (4) creating pure *M. glauca* plantations (MM) instead. The following issues are sought to be addressed: (1) How do different transformation patterns in *eucalypt* logging sites affect soil nutrient status after a rotation period? (2) To what extent will the planting of third‐generation pure plantations of EE negatively affect the soil fungal community diversity, composition, and stability of the forest floor? Can GS, EM, and MM modifications show improvement in degraded plantations compared to EE? (3) Could changes in soil fungal community composition and function be regulated by soil nutrient availability? What are the key drivers?

## 2 Materials and Methods

### 2.1 Study Area Description

The study site was selected in the state‐owned Qipo Forest Farm in Guangxi (22°68′ N, 108°20′E), which belongs to the southern subtropical monsoon climate with an annual precipitation of 1200–1300 mm and an annual mean temperature of approximately 21°C–22°C. Soil types were mainly ultisol, with rhodic paleudult as a modal profile (Staff 2010). The experiment was performed on an eucalypt plantation logging site located on the same slope (10 ha or more) in this forest farm. In 1981, the area was developed and planted with mixed fir *Cunninghamia lanceolata* × *Michelia macclurei* plantation, which was logged in 2004 and planted with pure *Eucalyptus urophylla* × *E. grandis* plantation with a density of 2500 plant·ha^−1^ in the same year. After harvesting the first generation of seedling‐planted eucalypt plantations in 2010, a new generation of sprouting plantation was cultivated. In 2016, after the logging of the second generation of plantations was completed, the logging site was left behind.

### 2.2 Experimental Design and Soil Sample Collection

In October 2016, the above‐selected slopes were divided into three equally sized blocks along the contour lines. Isolation zones were set up between the blocks, with the following four treatments randomized within each block: (1) inhibition of logging stump sprouting (41% glyphosate: water = 1:3) to form no‐forested areas dominated by grasses and shrubs (GS); (2) retaining healthy stump sprouting strips to form the third generation of *Eucalypt* plantations (EE); (3) based on treatment EE, the third generation of eucalypt plantations was thinned in May 2017 (the thinning ratio was 25%), and *M. glauca* seedlings with good growth vigor (50 cm ± 5 cm) were interplanted in the same month. Eventually, multi‐layered mixed plantations of *Eucalyptus urophylla* × *E. grandis* and *M. glauca* with the same stand density as that of the pure eucalypt plantations (EM) were formed; (4) on the basis of treatment EE, the eucalypt plantations were logged in May 2017, and pure *M. glauca* plantations (MM) were established. Compound fertilizer (N: P_2_O_5_: K_2_O = 15:6:9) was applied to the forested land for the first 3 years at 1.25 t ·ha^−1^ per application.

At the beginning of the experiment, three long‐term sample plots of 20 m × 20 m were set up in each replicate of the four treatments mentioned above. After one renovation cycle (based on the eucalypt rotation period), during the rainy season (August 2022) and dry season (March 2023) respectively, 8 soil samples (0–10 cm in depth) were randomly collected from each plot using a soil auger along the diagonal of each plot. After removing humus and debris, the samples were sieved and thoroughly mixed to form one replicate, with a total of 9 replicates obtained for each treatment. Then from each replicate, 2 g of soil was picked up into a 2 mL centrifuge tube, quickly deposited into liquid N, and stored in an ultra‐low temperature refrigerator at −80°C as soon as possible for DNA extraction. The remaining soil was separated equally, and one part was quickly transferred to a 4°C refrigerator for enzyme activity determination. In contrast, the other soil sample was air‐dried and used for chemical property determination. At the same time, nine undisturbed core soil samples from each treatment were collected using a core sampler (5 cm in diameter) for soil pH and moisture content measurements.

### 2.3 Soil Physicochemical Property Analysis

Soil gravimetric water content (SW) was determined using the desiccation method at 105°C (Cheng et al. 2023). Soil organic carbon (SOC) and dissolved organic carbon (DOC) content were determined using a potassium dichromate oxidation–external heating method and deionized water leaching method, respectively (Walkley 1935). Soil total nitrogen (TN) was determined using the Kjeldahl method. Nitrate nitrogen (${\mathrm{NO}}_{3}^{-}$_N) and ammonia nitrogen (NH+ 4_N) were determined using the 2 mol·L^−1^ KCl leaching‐indophenol blue colorimetric method and Ultraviolet–Visible spectrophotometry (RK 1999). Total phosphorus (TP) and available phosphorus (AP) content were measured using the molybdate–antimony–ascorbic acid colorimetric method (Cheng et al. 2023). The pH of each sample was determined in a soil–water (1:2.5) solution with an electronic pH meter (PHS‐3C, Shanghai INESA Scientific Instrument Co. Ltd., Shanghai, China) (Xu, Ren, et al. 2021).

### 2.4 Fungal DNA Amplicon Sequencing and Data Processing

Total microbial DNA was extracted from frozen soil samples using the CTAB method, and the purity and concentration were measured using NanoDrop2000 (Thermo Scientific, Wilmington, DE, United States). For fungal sequence analysis, the internal transcribed spacer region (ITS1) of the nuclear ribosomal RNA gene was amplified using primer pairs ITS1F (5′CTTGGTCATTTAGAGGAAGTAA 3′) and ITS2R (5′GCTGCGTTCTTCATCGATGC 3′) was used (Adams et al. 2013). Amplification was conducted in a T100 Thermal Cycler (Bio‐Rad, Hemel Hempstead, United Kingdom), and PCR products were extracted from 2% agarose gel and purified using an AxyPrep DNA Gel Extraction Kit (Axygen Biosciences, Union City, CA, USA) according to the manufacturer's instructions. The products were quantified using a Quantus Fluorometer (Promega, USA). Deep sequencing was performed on the Illumina Miseq PE300 platform (Illumina, CA, USA).

The optimized sequences after QC splicing were noise reduced to obtain amplicon sequence variants (ASVs) using the DADA2 plugin in the Qiime2 process (Callahan et al. 2016). The number of sequences in all samples was rarefied to 31,324 to minimize the impact of sequencing depth on subsequent alpha and beta diversity analyses. This procedure resulted in an average Good's coverage of 99.8% per sample. Species classification of ASVs was based on the UNITE v8 gene database (https://unite.ut.ee/) using the Naive Bayes classifier in Qiime2 (Bolyen et al. 2019). Then, Mothur (1.30.2, https://www.mothur.org/wiki/Download_mothur) was used to calculate the Shannon, Chao1, and Pielou_e indices. The nutritional types and functional groups (functional guilds) of the soil fungal community were predicted by running the “FUNGuild” algorithm. Only the “highly probable” and “probable” confidence levels were retained in this analysis to avoid over‐interpreting the functional guilds (Nguyen et al. 2016).

### 2.5 Fungal Co‐Occurrence Network Analysis

Network relationships between fungal taxa were constructed using the WGCNA package (Langfelder and Horvath 2008). Using data from a total of 36 soil samples across 4 treatments in each season, this study constructed seasonal co‐occurrence networks. ASVs with an average relative abundance (RA) greater than 0.01% in the soil were selected for Spearman correlation analysis, and 0.86 was used as a suitable similarity threshold for the test region using random matrix theory (Luo et al. 2006). The igraph package, via its subgraph functions, was employed to extract edge data, node data, and topological features of the fungal networks for each treatment. Gephi (https://gephi.org/) was used for the visualization of the network graphs (Qiu et al. 2021). The fungal network topological features for each sample were implemented in the subgraph function to validate the network complexity via the “igraph” and “multifunc” packages (Ma et al. 2016). Natural connectivity was used to estimate the structural robustness of soil fungal networks and determine the stability of fungal networks (Peng and Wu 2016).

### 2.6 Statistical Analysis

One‐way ANOVA (*p* < 0.05) based on Duncan's test was performed to test differences in soil physicochemical properties, microbial diversity, RA of dominant fungi, topological characteristics of fungal networks, and functional guilds. In order to visualize the overall differences in fungal community structure between treatments, non‐metric multidimensional scaling (NMDS) based on the Bray–Curtis distance and permutational multivariate analysis of variance (PERMANOVA) was used to determine the differences in the composition of fungal communities between any two treatments. Mantel tests were used to investigate the effect of soil physical and chemical properties on the structural and functional composition of fungal communities. All the above data analyses were performed in R.4.0.3. Additionally, redundancy analysis (RDA, Canoco5, http://www.canoco5.com/) and Spearman correlation analysis were utilized to determine the relationship between soil physicochemical properties and fungal community structure, as well as functional profiles.

## 3 Results

### 3.1 Changes in Soil Physicochemical Properties

**FIGURE 1:**
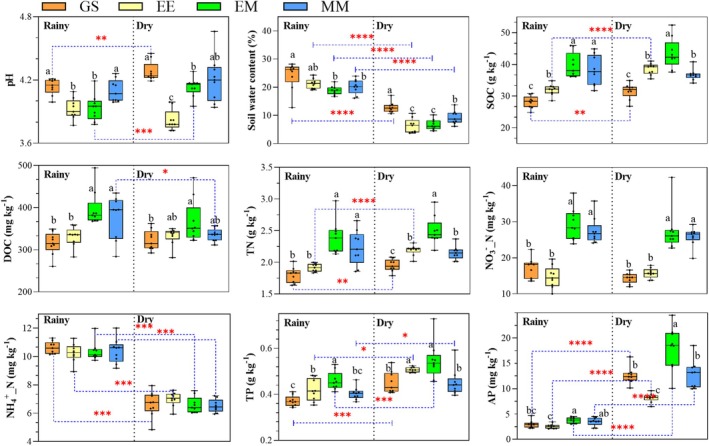
Changes in soil physicochemical properties in four logging site management patterns, including pH, soil–water content (SW), soil organic carbon (SOC), dissolved organic carbon (DOC), total nitrogen (TN), nitrate nitrogen (${\mathrm{NO}}_{3}^{-}$_N), ammonium nitrogen (NH+ 4_N), total phosphorus (TP), and available phosphorus (AP), in both rainy and dry seasons. GS: Unforested sites covered with grasses and shrubs after planting two generations of eucalypt plantations; THE: The third generation of pure eucalypt plantations; EM: *E. urophylla* × *M. glauca* mixed plantations; MM: Monoculture *M. glauca* plantations. Different lowercase letters above the columns indicate significant differences in soil properties between treatments at the *p* < 0.05 level. *, **, ***, and **** represent *p* < 0.05, *p* < 0.01, *p* < 0.001, and *p* < 0.0001, respectively.

The different transformation patterns on eucalypt logging sites caused significant differences in soil physico‐chemical properties at the end of a transformation cycle during both the dry and rainy seasons (measured in terms of eucalypt rotation period; Figure 1; *p* < 0.05). More specifically, SOC, DOC, TN, ${\mathrm{NO}}_{3}^{-}$_N, and AP contents were significantly higher in mixed plantation EM and pure *M. glauca* plantation MM than those in the unforested area GS and pure eucalypt plantation EE in the rainy season. At the same time, only EM had higher SOC, DOC, TN, ${\mathrm{NO}}_{3}^{-}$_N, TP, and AP contents than those in the GS, EE, and MM treatments during the dry season. Meanwhile, the soil pH in eucalypt plantations (EE and EM) was significantly lower than that of GS and MM in both seasons, especially in EE during the dry season. Furthermore, the forestland treatments (EE, EM, and MM) showed lower soil moisture content compared to GS. Soil SOC, TN, TP, and AP contents tended to increase to varying degrees from the rainy season to the dry season, while SW and NH+ 4_N significantly decreased (Figure 1; *p* < 0.05).

### 3.2 Fungal Community Diversity and Network Complexity

Although fungal diversity did not differ significantly among treatments, the transformation patterns significantly affected soil fungal community richness and evenness (Figure S1). In particular, the fungal richness indices (Chao1 and Sobs) of the forested treatments (EE, EM, and MM) were significantly higher than those of the non‐forested GS in both the rainy and dry seasons, with the mixed plantation EM showing a significant advantage over the pure plantations (EE and MM) (Figure S1).

**FIGURE 2:**
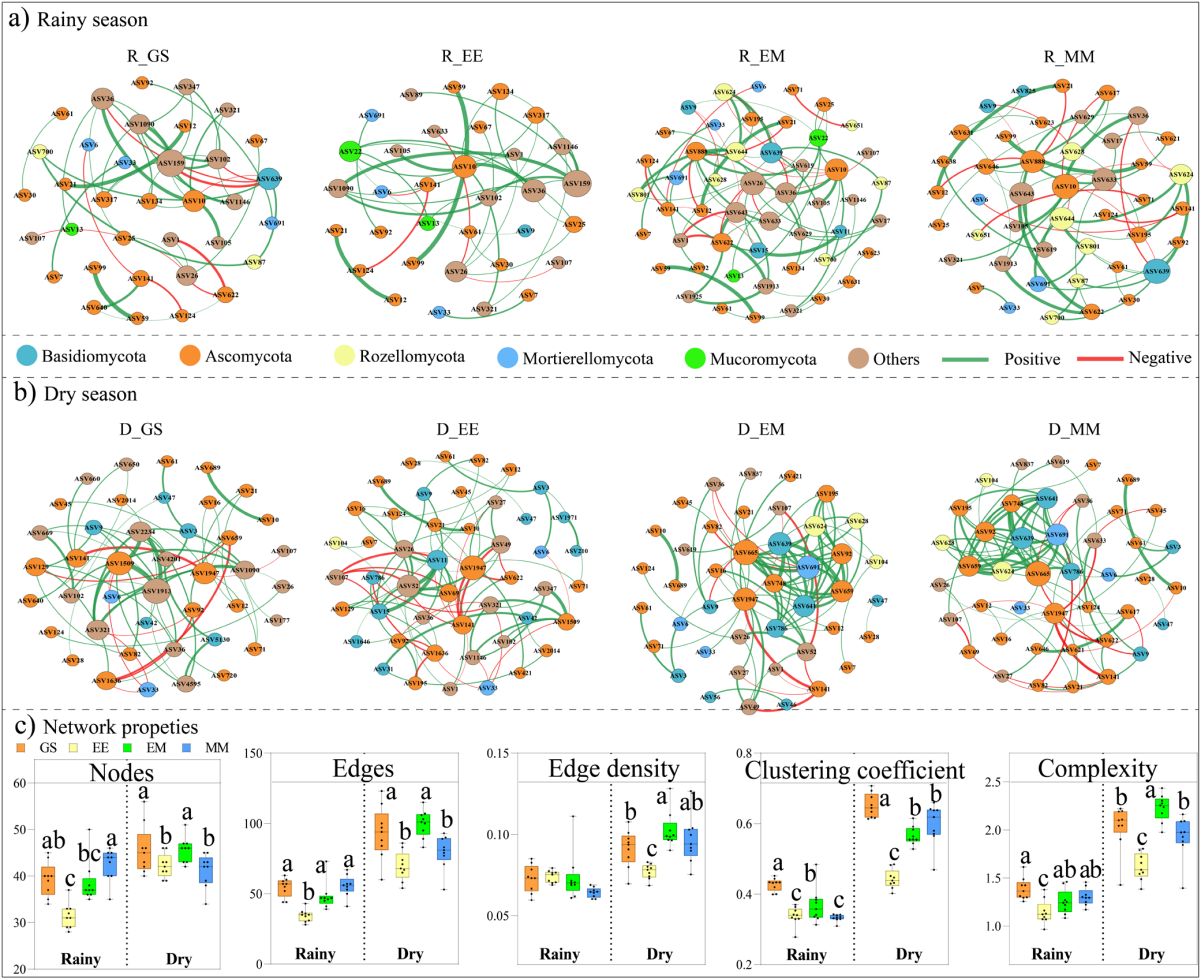
Soil fungal co‐occurrence network in the rainy season (a) and dry season (b) in different management patterns. Green and red edges indicate significant positive and negative correlations between nodes, respectively (Spearman, *p* < 0.05). EE, the third generation of pure eucalypt plantations; EM, *E. urophylla* × *M. glauca* mixed plantations; GS, unforested sites covered with grasses and shrubs after planting two generations of eucalypt plantations; MM, monoculture *M. glauca* plantations. (c) Co‐occurrence network topological characteristics, including nodes, edges, edge density, clustering coefficient, and complexity. Different lowercase letters above the columns indicate significant differences between treatments at the *p* < 0.05 level.

Fungal co‐occurrence network topological properties can reflect the complexity and robustness of the network structure. In this study, the eucalypt continuous planting treatment (EE) significantly reduced fungal network nodes, edges, edge densities, and clustering coefficients compared to GS, EM, and MM. Compared with EE, soil fungal network complexity increased by 21.2%, 9.43%, and 14.07% for GS, EM, and MM treatments in the rainy season and by 37.40%, 42.72%, and 35.85%, respectively, in the dry season (Figure 2a–c). Additionally, the natural connectivity of EM and MM was obviously higher than that of EE and GS by gradually removing the network nodes (Figure S2), reflecting the improvement effect of EM and MM on the complexity and robustness of soil fungi on the forest floor.

### 3.3 Fungal Community Structure and Composition

**FIGURE 3:**
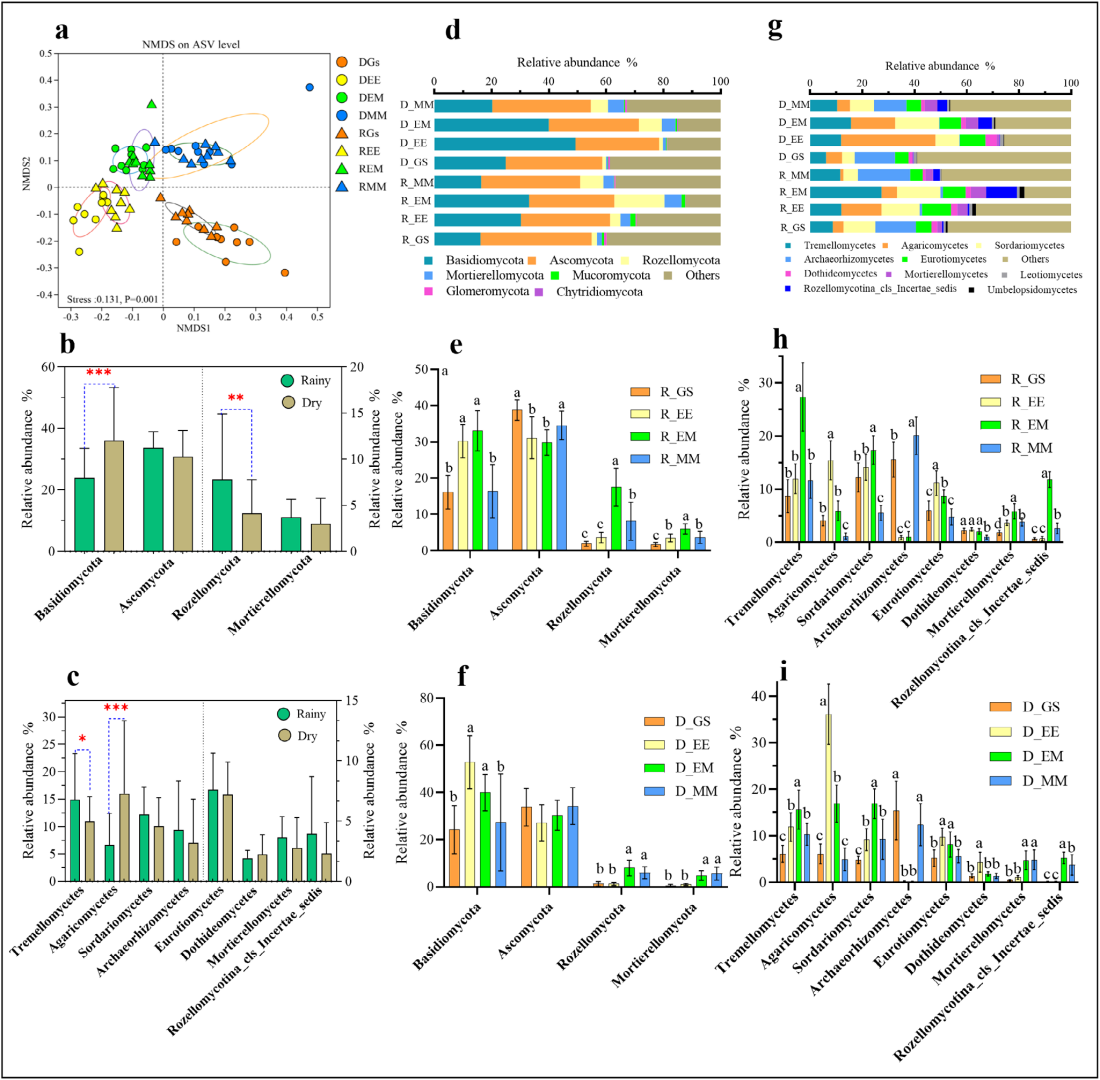
Non‐metric multidimensional scaling ordination (NMDS) of fungal communities based on the ASV level (a). Seasonal variation induced differences in the RA of soil fungal phyla (b) and class (c). Different management patterns induced variations in the RA of soil fungal phyla (d–f) and phyla (g–i) during the dry and rainy seasons. EE, the third generation of pure eucalypt plantations; EM, *E. urophylla* × *M. glauca* mixed plantations; GS, unforested sites covered with grasses and shrubs after planting two generations of eucalypt plantations; MM, Monoculture *M. glauca* plantations. Different lowercase letters above the columns indicate significant differences between treatments at the *p* < 0.05 level.*, **, ***, and **** represent *p* < 0.05, *p* < 0.01, *p* < 0.001, and *p* < 0.0001, respectively.

The logging site transformation patterns caused more significant differences in soil fungal community structure and dominant fungal RAs compared to the sampling seasons, as verified by subsequent PERMANOVA analyses (Figure 3a–i and Table S1; *p* < 0.01). Additionally, most of the fungal taxa RAs differed significantly among the treatments despite similar dominant fungal species composition (Figure 3d–i; *p* < 0.01). For example, the dominant soil fungi in this experimental area at the phylum level mainly included Basidiomycota, Ascomycota, Rozellomycota, Mortierellomycota, Mucoromycota, Glomeromycota, and Chytridiomycota, accounting for 60.18%–87.76% of the total fungal sequences. Among them, the RAs of Basidiomycota were higher in EE and EM soils than in GS and MM soils. At the same time, the Ascomycota RAs showed the opposite trend and reached a significant level during the rainy season. Moreover, the RAs of Rozellomycota and Mortierellomycota were markedly higher in both EM and MM than in GS and EE (except for Mortierellomycota in MM during the rainy season) (Figure 3e,f; *p* < 0.01).

Furthermore, regarding the class level, EM and MM enhanced the RAs of Mortierellomycetes and Rozellomycotina_cls_Incertae_sedis compared to GS and EE, especially in the dry season. At the same time, EM significantly enhanced the RAs of Tremellomycetes and Sordariomycetes compared to GS, EM, and MM during both dry and rainy seasons. In contrast, EE dramatically elevated the RA of Agaricomycetes. Non‐negligibly, Archaeorhizomycetes RA was higher in GS and MM than in EE and EM. In contrast, the opposite trend was observed in the RA of Eurotiomycetes (Figure 3h,i; *p* < 0.01).

### 3.4 Soil Fungal Community Functional Guilds

**FIGURE 4:**
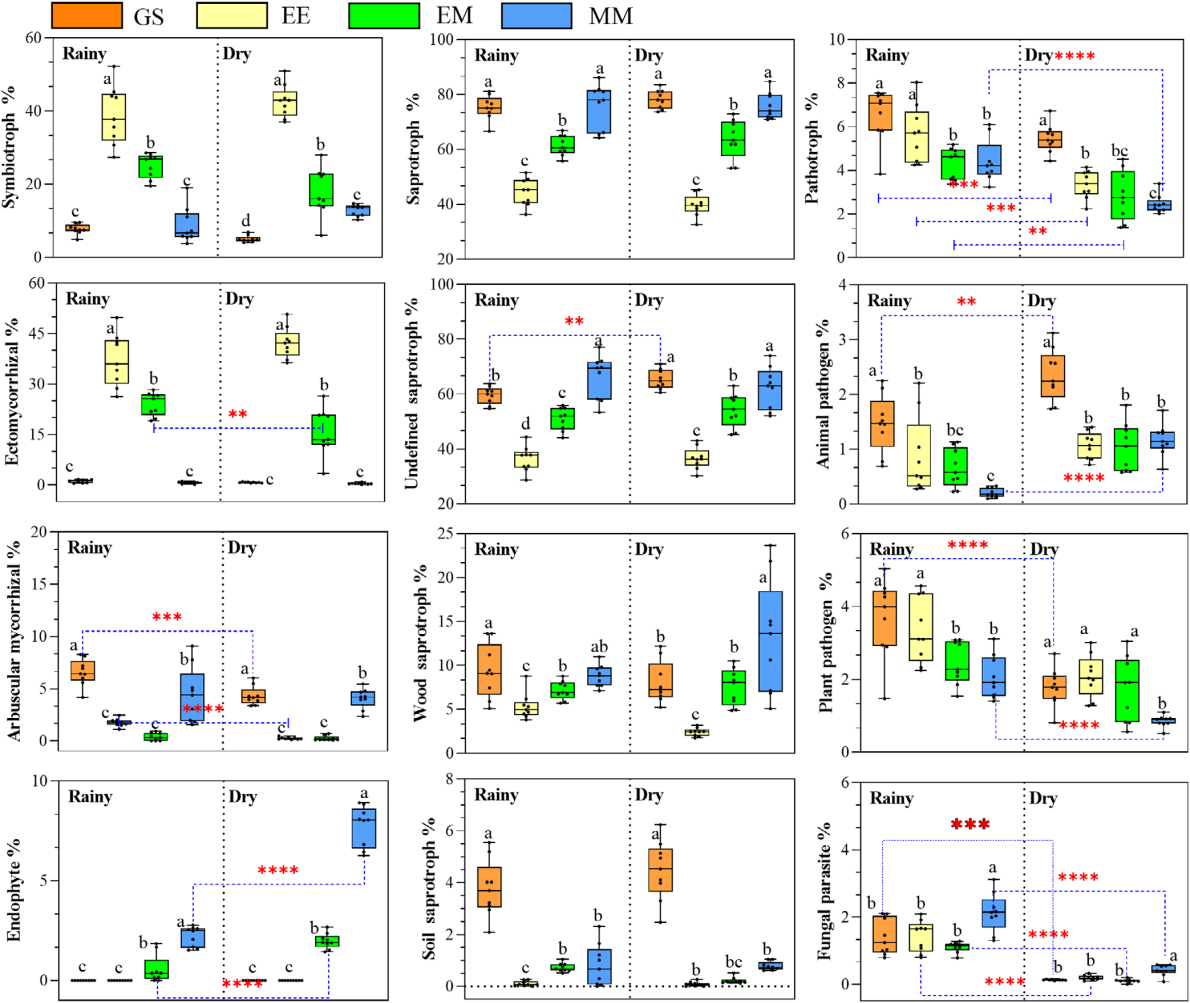
Distribution of functional guilds of soil fungal communities as inferred using FUNGuild for different management patterns in the dry and rainy seasons. EM, *E. urophylla* × *M. glauca* mixed plantations; GS, unforested sites covered with grasses and shrubs after planting two generations of eucalypt plantations; MM, monoculture *M. glauca* plantations; THE, the third generation of pure eucalypt plantations. Different lowercase letters above the columns indicate significant differences in soil properties between treatments at the *p* < 0.05 level. *, **, ***, and **** represent *p* < 0.05, *p* < 0.01, *p* < 0.001, and *p* < 0.0001, respectively.

Functionally, the distribution patterns of the different trophic fungi differed significantly among the transformation patterns (Figure 4). The vast majority of fungal ASVs were identified in three major categories of trophic fungi, mainly saprophytic fungi (32.65%–87.51%), symbiotic fungi (3.79%–52.21%), and pathological fungi (2.02%–8.05%). In terms of symbiotic fungi, the RAs of total symbiotic fungi and overwhelmingly dominant ectomycorrhizal (ECM) fungi were significantly higher in EE than in GS and MM, and the RAs of these fungi in EM were between EE and MM. Meanwhile, the treatments (EE and EM) that removed eucalypts from the logging sites showed marked reductions in arbuscular mycorrhizal fungi. The RA of saprophytic fungi showed the opposite distribution pattern to that of total saprophytic fungi among treatments, with EE significantly decreasing the relative abundance of saprophytic fungi. The RA of saprophytic fungi showed the opposite distribution pattern to that of total saprophytic fungi among treatments, with EE significantly decreasing the RA of saprophytic fungi. This study also found that the RAs of total pathogenic fungi were lower or significantly lower in EM and MM than in GS and EE in both rainy and dry seasons. At the same time, the RAs of total pathological fungi, plant pathogens, and fungal pathogens in the dry season were all lower than those observed in the rainy season.

### 3.5 Factors Influencing Fungal Community Structure and Function

**FIGURE 5:**
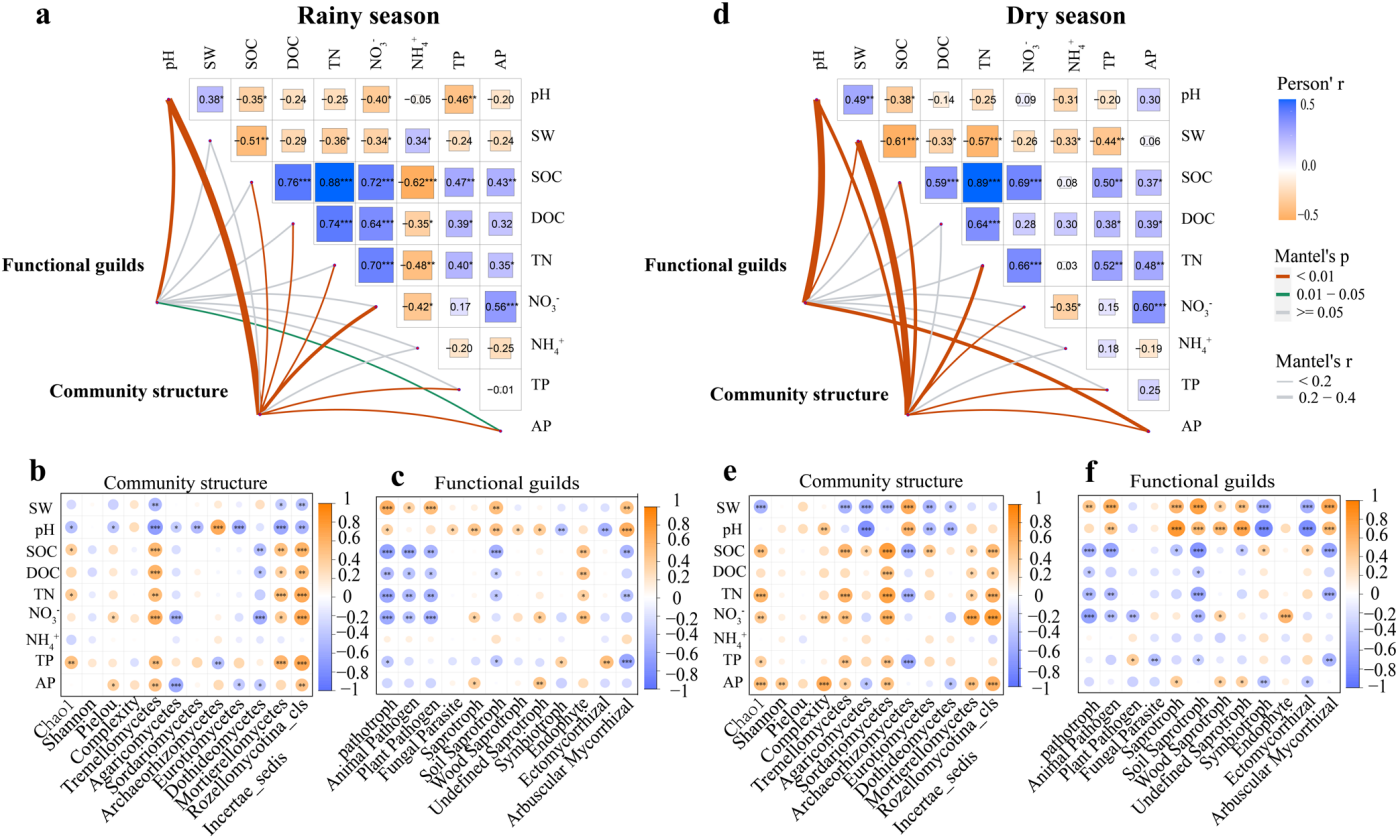
Soil abiotic factors influencing fungal community structure and function for different management patterns in the rainy (a) and dry (d) seasons. Correlation between soil fungal diversity, dominant class‐level fungi, and soil abiotic factors in the rainy (b) and dry (e) seasons. Correlation between soil fungal functional guild and soil abiotic factors in the rainy (c) and dry (f) seasons. AP, available phosphorus; DOC, dissolved organic carbon; NH+ 4, ammonium nitrogen; ${\mathrm{NO}}_{3}^{-}$, nitrate nitrogen; SOC, soil organic carbon; SW, soil–water content; TN, total nitrogen; TP, total phosphorus.

**FIGURE 6:**
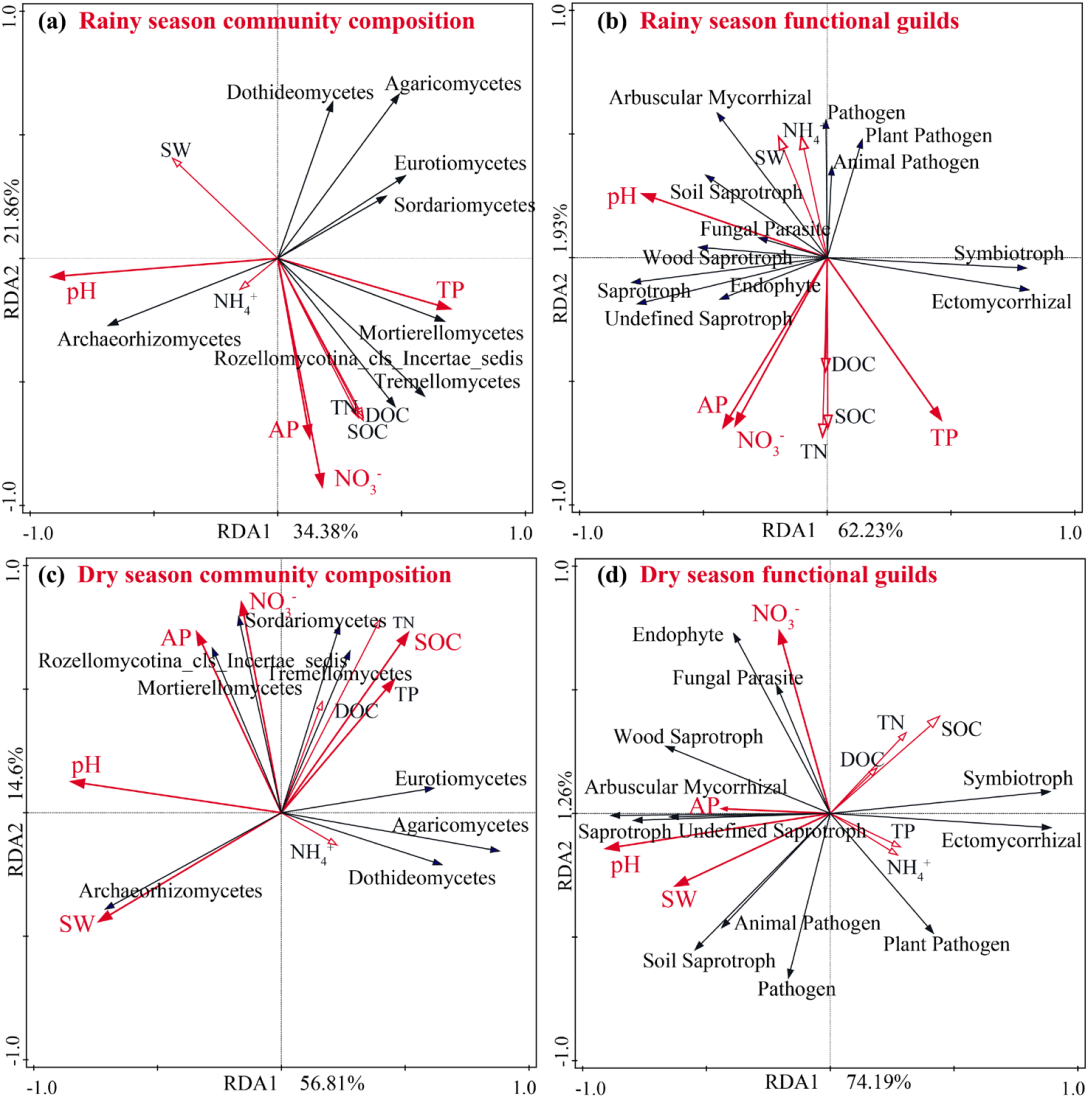
Effect of soil abiotic factors on fungal community composition and function. Redundancy analysis reveals the relationship between soil abiotic factors and fungal community composition in the rainy season (a) and dry season (c) or fungal trophic patterns in the rainy season (b) and dry season (d).

Soil fungal community composition and functional guilds distribution were driven by pH, ${\mathrm{NO}}_{3}^{-}$_N, and AP content in both the rainy and dry seasons and were also significantly affected by SW in the dry season (Figures 5 and 6). Among them, the RAs of Tremellomycetes, Mortierellomycetes, and Rozellomycotina_cls_Incertae_sedis in both seasons, as well as Sordariomycetes in the dry season, were positively correlated with SOC, DOC, TN, ${\mathrm{NO}}_{3}^{-}$_N, TP, and AP, and negatively correlated with SW. At the same time, the opposite relationship was observed for Dothideomycetes (rainy season samples) as well as Archaeorhizomycetes (dry season samples) (Figure 5b,e). Regarding functional composition, soil pH, ${\mathrm{NO}}_{3}^{-}$_N, and AP content were positively correlated with RAs of soil wood saprotroph and undefined saprotroph and negatively correlated with RA of ectomycorrhizal fungi. In contrast, the RA of pathogenic fungi was negatively correlated with SOC, DOC, and most nutrient contents and positively correlated with SW and pH (Figure 5c,f).

## 4 Discussion

### 4.1 Response of Soil Physicochemical Properties to Logging Site Transformation Patterns

Plantation management strategies significantly affect soil physicochemical properties (Zhou et al. 2020). In this study, EM and MM significantly enhanced soil nutrient availability, such as DOC, ${\mathrm{NO}}_{3}^{-}$_N, and AP content, compared with GS and EE after six years of transformation, and in particular, EM also enhanced SOC, TN, and TP content dramatically. This result may be attributed to the differences in nutrient uptake and return characteristics of dominant tree species or vegetation in different ecosystems caused by transformation patterns (Bai et al. 2023; Li, Hong, et al. 2023). Multigeneration continuous planting management of EE captured a large amount of effective soil nutrients and induced the accumulation of low‐quality litter, which caused a large negative impact on litter decomposition processes and nutrient returns (Zhou et al. 2020; Xu, Li, et al. 2021). Meanwhile, the reduction in available soil nutrients in GS may be attributed to surface runoff resulting from unforested sloped lands (Zheng et al. 2005). However, mixed plantation EM, as well as treatment MM formed with *M. glauca* as a rotation species, not only reduced fast‐growing tree densities and improved the microenvironment (e.g., increased understory light and forest penetrating rainfall) but also enriched nutrient return pathways, that is, increased litter quality and root death diversity (Trogisch et al. 2015; Latterini et al. 2023). These factors resulted in a significant increase in soil nutrient effectiveness in EM and MM. Remarkably, the continuous planting of eucalypts caused a significant decrease in soil pH, especially in the EE treatment during the dry season, which is in line with previous studies (Zhu et al. 2019; Xu, Li, et al. 2021). This result may be attributed to the accumulation of organic acids during the decomposition of low‐quality litter and the high application of inorganic fertilizers (Rhoades and Binkley 1996). Encouragingly, our transformation patterns GS, EM, and MM all obviously alleviated the soil acidification trend compared with EE.

### 4.2 Implications of Logging Site Transformation Patterns on Fungal Diversity and Ecological Network Complexity

Differences in soil nutrient availability and vegetation types induced by the logging site transformation patterns shaped the distributional characteristics of fungal community diversity (Geng et al. 2023; He et al. 2024). In this study, soil fungal richness indices were dramatically lower in unforested treatments (GS) than in forested sites (EE, EM, MM). However, the differences in fungal diversity and dry season evenness indices were not significant (Figure S1). This result may be related to the absence of ecological niches for fungal colonization (Hartmann et al. 2013). Soil fungal colonization strongly depends on their hosts. Compared to non‐forested sites, vigorous root growth in forest soils promotes both root secretion accumulation and soil aeration, which in turn contributes to fungal metabolic activity and richness (Wang, Zhang, et al. 2019; Zhang, Feng, et al. 2023). In contrast, soil fungal richness and evenness in EM showed clear advantages over pure plantations (EE, MM) (Figure 5), providing more fungal‐mediated benefits to plant and soil ecosystems (Qin et al. 2024). This result may be attributed to the significant positive correlation between fungal richness and evenness with SOC, DOC, TN, ${\mathrm{NO}}_{3}^{-}$_N, TP, and AP (Figure 5b,e). On the one hand, the enhancement of soil active C and nutrient content in mixed plantations effectively promotes the rapid colonization of different microbial populations and realizes the harmonious coexistence of microbial groups by weakening the dominant position of the most competitive species (Wu et al. 2019). On the other hand, the increased diversity of litter and root systems provided diversified habitats for more species of microorganisms to colonize (Yang et al. 2019).

The topological characteristics of ecological networks reflect microbial community complexity and robustness in response to environmental changes (Neutel et al. 2007; Luo et al. 2023). As this study found here, EE significantly reduced fungal ecological network complexity and robustness (Figure 2c and Figure S2), possibly due to soil nutrient imbalance and limited microbial availability caused by continuous planting of monoculture tree species (Cheng et al. 2023), which resulted in a large number of fungi going dormant, thus simplifying the fungal co‐occurrence network (Li, Xu, et al. 2023). By contrast, the advantages of mixed plantation EM in terms of nodes, edges, edge density, network complexity, and robustness in the dry season were obvious, which was also confirmed in previous studies (Pereira et al. 2018). Qin et al. (2024) concluded that the mixed pattern of eucalypts and native species significantly increased the co‐occurrence network complexity and stability of soil C, N, and P transformation genes, emphasizing the rapid response of functional microorganisms to soil nutrient cycling processes in mixed plantations.

### 4.3 Effects of Logging Site Transformation Patterns on Soil Fungal Community Structure and Functions

Although the soil fungal compositions were similar among the different logging site transformation patterns, the community structure varied significantly, with the dominant vegetation on the logging sites (Figure 3). In the study area, soil fungi with an average RA greater than 1% belonged to Basidiomycota, Ascomycota, Rozellomycota (formerly known as Cryptomycota), and Mortierellomycota, which was similar to the composition of forest soil fungi on a global scale (Tedersoo et al. 2014). In addition, Basidiomycota fungi were enriched in the soils of EE and EM formed after sprout regeneration of stumps. This result may be explained by the low‐quality substrate rich in lignin, cellulose, and phenolic acid compounds accumulated in eucalypt *plantation*s over many years, which creates conditions for the colonization of saprophytic Basidiomycota (Allison et al. 2007). Meanwhile, the symbiotic relationship between eucalypt root systems and symbiotic Basidiomycota provides a guarantee of eucalypt nutrient uptake (Read and Perez‐Moreno 2003; Xu, Li, et al. 2021). At the same time, the high proportion of Ascomycetes fungi in unforested GS and rotation‐planted plantation MM may be attributed to significant changes in the composition of dominant vegetation. Relevant research indicates that Ascomycota fungi tend to play an important role in the early stages of community succession or in soils that have experienced substantial disturbances (Ammitzboll et al. 2021).

At the class level, Agaricomycetes fungi were highly enriched in EE soil compared to GS, EM, and MM and showed a negative correlation with ${\mathrm{NO}}_{3}^{-}$_N and AP (Figure 3). This result was reflected in previous studies, where a significant number of fungi belonging to the Agaricomycetes tended to form a mutually beneficial symbiosis with the root system of nutrient‐poor continuous *eucalypt* plantations (Read and Perez‐Moreno 2003; Wu et al. 2019; Xu et al. 2022). Whereas Mortierellomycetes and Rozellomycotina_cls_Incertae_sedis were abundant in EM and MM soils, Tremellomycetes and Sordariomycetes were significantly enriched in EM soils. These fungi were all positively correlated with soil SOC, DOC, and most nutrient indices, which may be attributed to their extensive capacity for cellulose and lignin degradation (Wilhelm et al. 2017; Wu et al. 2019; Li, Xu, et al. 2023; Błońska et al. 2024) and high utilization efficiency of simple carbohydrates (Osorio and Habte 2013; Wang et al. 2017). In this study, the RA of Archaeorhizomycetes belonging to the Ascomycota was also found to be significantly higher in the GS and MM than in the EE and EM treatments. Given that the specific ecological roles of this class of fungi are uncertain (Pinto‐Figueroa et al. 2019), we hypothesize that differences in vegetation types induced by various management practices dominate the divergent distributions of this class of species.

The distribution of fungal functional guilds largely reflected the variability in soil nutrient availability among the logging site transformation patterns, and soil nutrient deprivation due to high‐intensity silvicultural patterns made eucalypt growth highly dependent on mycorrhizal fungi (Read and Perez‐Moreno 2003; Castano et al. 2019). This result was also observed in the present study, in which ectomycorrhizal fungi were significantly enriched in EE (Figure 4). The possible reason lies in that, as a dual mycorrhizal model plant, the new generation of eucalypt budding plantations can continuously supply organic matter for the colonization of underground mycorrhizal fungi (Adjoud‐Sadadou and Halli‐Hargas 2017). Moreover, ECM fungi provide important nutrient support to meet host needs by efficiently absorbing soil resources through extended mycelial networks and degrading complex organic N compounds by secreting extracellular enzymes (Gao et al. 2022). This mutualistic relationship between continuously planted eucalypt systems and ECM fungi has been found in several *Eucalyptus* species (Zheng et al. 2017; Castano et al. 2019; Xu, Li, et al. 2021).

Compared with EE, the other modifications significantly enhanced the RA of the soil saprophytic fungi (Figure 4). This may be attributed to the partial removal of eucalypts from the plantation (EM) or their complete removal (GS, MM), both of which have disrupted the balance of the original symbiotic relationship dominated by ectomycorrhizal (ECM) fungi. As a result, more ecological niches are provided for soil saprophytic fungi, strengthening the relationship between saprophytic fungi, which are proficient in decomposing plant litter and soil organic matter, and the stability of the ecosystem (Johnson 2010; Geng et al. 2023). Recent studies have found that the RA of saprophytic fungi was significantly and positively correlated with soil fertility (Li, Xu, et al. 2023). This result was also confirmed in the present study, where undefined saprophytic and wood saprophytic fungi were significantly enriched in EM and MM soils, and their RAs were positively correlated with soil‐effective nutrients (Figures 1 and 5). However, the elevated proportion of saprophytic fungi did not increase the soil nutrient status of GS, which may be attributed to the scouring of topsoil by slope runoff (Zheng et al. 2005). Meanwhile, this study found that EM and MM significantly reduced the RA of total pathogenic fungi, probably because these microorganisms lacked saprophytic life stages, and the replacement of dominant vegetation prevented them from surviving due to the lack of original living hosts (Bailey and Duczek 1996).

### 4.4 Key Soil Factors Driving Changes in Soil Fungal Communities

Soil nutrient availability and key physical properties determine the distribution pattern of the soil fungal community structure and functional guilds (Liu et al. 2018; Canini et al. 2019; Xie and Yin 2022). Similarly, in this study, the distribution of fungal community composition and functional guilds in both seasons was jointly driven by pH, ${\mathrm{NO}}_{3}^{-}$_N, and AP contents and further significantly influenced by SW in the dry season (Figures 5 and 6). These environmental factors may directly shape the fungal community by influencing enzyme activities and mycorrhizal colonization (Deacon 2006; Glassman et al. 2017; Canini et al. 2019; Wu et al. 2019). As confirmed by this study and other related research, multigeneration continuous eucalypt pure plantations decreased soil pH and soil nutrient bioavailability (AP and ${\mathrm{NO}}_{3}^{-}$_N). Still, they increased the RA of soil symbiotic fungi. They had the lowest proportion of saprophytic fungi, whereas mixed and rotational plantations were effective in increasing the RA of saprophytic fungi and soil nutrient availability (Read and Perez‐Moreno 2003; Li, Xu, et al. 2023). Meanwhile, this study found that SW notably affected changes in the fungal community structure only during the dry season. This result may be due to the fact that sufficient rainfall during the rainy season in southern China did not affect plantation growth or microbial reproduction (Hua et al. 2021). However, the dry season significantly reduced SW (Figure 1), coupled with competition for soil moisture by vegetation growth, making SW an important limiting factor for fungal community development in the dry season (Wang, Du, et al. 2019).

Nevertheless, while functional predictions derived from amplicon sequencing exhibit a robust correlation with metagenomic profiles, supplementary experimental validation—such as shotgun metagenomics or targeted functional gene sequencing—remains warranted (Winter 2021). Furthermore, it is critical to recognize that distinct isolates of the same microbial species can display marked variations in their biochemical traits (da Silva et al. 2012). Additionally, the presence of microbial DNA in natural ecosystems does not equate to the viability or metabolic activity of the corresponding organisms (Joergensen et al. 2024).

## 5 Conclusions

In conclusion, this study investigated the combined effects of eucalypt logging site transformation patterns and seasonal variations on soil fungal community diversity, composition, and function. This study identified the key soil factors influencing fungal community changes. Specifically, differences in vegetation types among the four transformation patterns affected fungal communities more than seasonal variations, and soil pH, SW, ${\mathrm{NO}}_{3}^{-}$_N, and AP content were the key driving factors influencing fungal community diversity, structural composition, and function. After a 6‐year transformation period, SOC, ${\mathrm{NO}}_{3}^{-}$_N, and AP contents were significantly lower in the unforested sites (GS) as well as in the eucalypt pure plantations (EE) than in the EM and MM, which may be attributed to their regulation of the understory microenvironment. Correspondingly, the continuous planting pattern (EE) significantly increased plantation dependence on mycorrhizal fungi and simplified the complexity of the fungal co‐occurrence network. Although GS elevated the RA of saprophytic fungi, the unforested conversion on slopes caused significant negative impacts on soil nutrient availability and fungal richness. Compared with these two models, EM and MM enhanced SOC, nutrient effectiveness, and the complexity and stability of fungal networks and obviously inhibited soil acidification and the spread of pathogenic fungi. Overall, this study concludes that adopting reasonable management patterns for eucalypt logging sites can help repair soil quality and provide breeding habitats for diverse microorganisms, which is important for maintaining the sustainable development of plantation forest ecosystems.

## Author Contributions

**Yuxing Xu:** formal analysis (equal), investigation (equal), methodology (equal), visualization (equal), writing – original draft (equal). **Zhichao Wang:** investigation (equal), methodology (equal). **Runxia Huang:** formal analysis (equal), investigation (equal), methodology (equal). **Wankuan Zhu:** formal analysis (equal), investigation (equal), methodology (equal). **Apeng Du:** funding acquisition (equal), project administration (equal), resources (equal), validation (equal), writing – review and editing (equal). **Chao Li:** investigation (equal), software (equal), supervision (equal), validation (equal), writing – review and editing (equal).

## Conflicts of Interest

The authors declare no conflicts of interest.

## Supporting information


**Table S1:** Pairwise comparison of soil fungal community structure between GS, EE, EM, and MM in the PERMANOVA analysis in the dry and rainy seasons.
**Figure S1:** Changes in soil fungal diversities in four logging sites management patterns, including Shannon indes, Chao1index, sobs, and Pielou_e index, in rainy and dry seasons. GS: unforested sites covered with grasses and shrubs after planting two generations of eucalypt plantations; EE: the third generation of pure eucalypt plantations; EM: *E. urophylla* × *M. glauca* mixed plantations; MM: monoculture *M. glauca* plantations. Different lowercase letters above the columns indicate significant differences between treatments at the *p* < 0.05 level. ****p < 0.0001.
**Figure S2:** Natural connectivity of soil microbial networks in relation to the number of removal nodes in rainy season (a) and dry season (b) in different management patterns.

## Data Availability

The raw reads were deposited into the NCBI Sequence Read Archive (SRA) database (PRJNA1245647).
