# Supplementary material for: Soil Nutrient Availability Drives Fungal Community Structure and Function During the Transformation of Eucalypt Plantations Logging Sites in Southern China
Source: Ecol Evol. 2025 Nov 12;15(11):e72263. doi: 10.1002/ece3.72263 (PMC12611881; doi:10.1002/ece3.72263)
Supplement: Supplementary file 1 — Table S1: Pairwise comparison of soil fungal community structure between GS, EE, EM, and MM in the PERMANOVA analysis in the dry and rainy seasons. Figure S1: Changes in soil fungal diversities in four logging sites management patterns, including Shannon indes, Chao1index, sobs, and Pielou_e index, in rainy and dry seasons. GS: unforested sites covered with grasses and shrubs after planting two generations of eucalypt plantations; EE: the third generation of pure eucalypt plantations; EM: E. urophylla × M. glauca mixed plantations; MM: monoculture M. glauca plantations. Different lowercase letters above the columns indicate significant differences between treatments at the p < 0.05 level. ****p < 0.0001. Figure S2: Natural connectivity of soil microbial networks in relation to the number of removal nodes in rainy season (a) and dry season (b) in different management patterns. [file ECE3-15-e72263-s001.docx]

Table. S1. Pairwise comparison of soil fungal community structure between GS, EE, EM, and MM in the PERMANOVA analysis in the dry and rainy seasons.

|  | DGS |  | DEE | DEM |  |  | RGS |  | REE | REM |
| --- | --- | --- | --- | --- | --- | --- | --- | --- | --- | --- |
| DEE | *** |  |  |  |  | REE | *** |  |  |  |
| DEM | *** |  | *** |  |  | REM | *** |  | *** |  |
| DMM | *** |  | *** | *** |  | RMM | *** |  | *** | *** |
|  |  |  |  |  |  |  |  |  |  |  |
|  | DGS |  |  | DEE |  |  | DEM |  |  | DMM |
| RGS | ** |  | REE | *** |  | REM | *** |  | RMM | * |


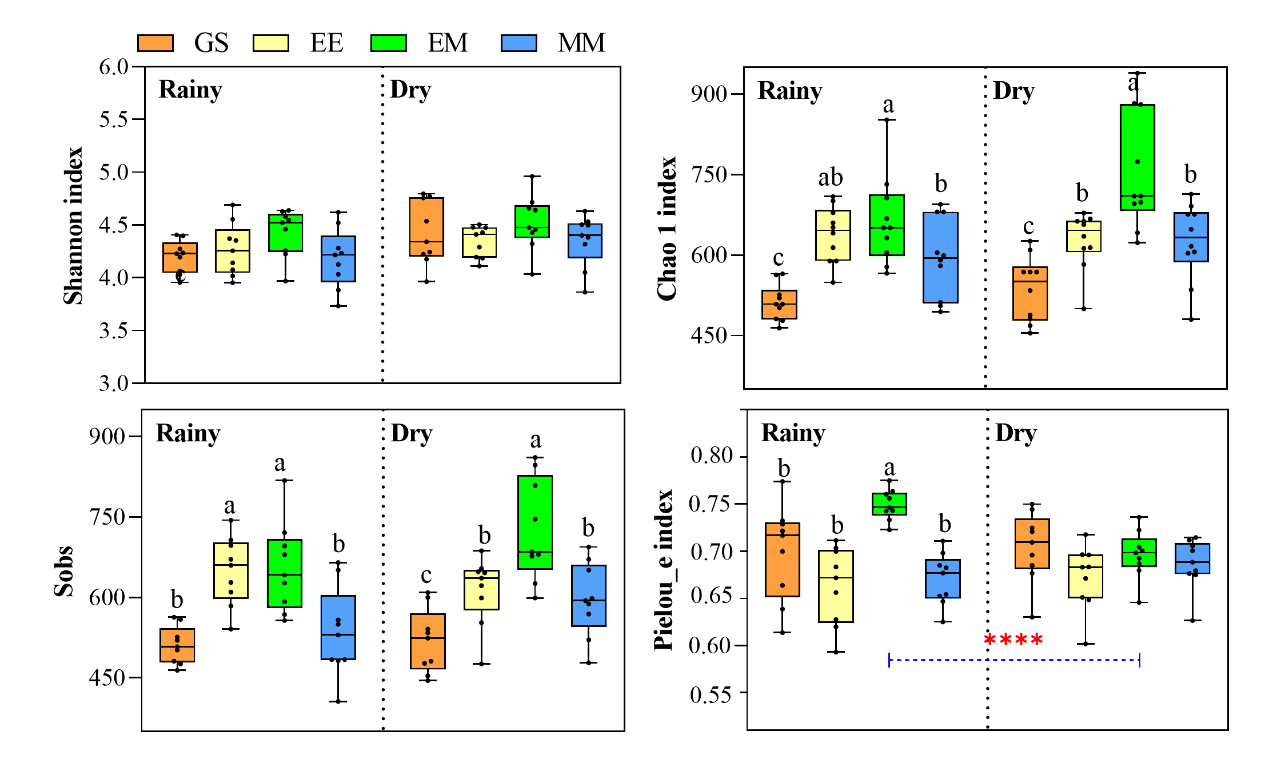


Fig. S1. Changes in soil fungal diversities in four logging sites management patterns, including Shannon indes, Chao1index, sobs, and Pielou_e index, in rainy and dry seasons. GS: unforested sites covered with grasses and shrubs after planting two generations of eucalypt plantations; EE: the third generation of pure eucalypt plantations; EM: *E. urophylla* × *M. glauca* mixed plantations; MM: monoculture *M. glauca* plantations. Different lowercase letters above the columns indicate significant differences between treatments at the *P* < 0.05 level. **** represent *P* < 0.0001.


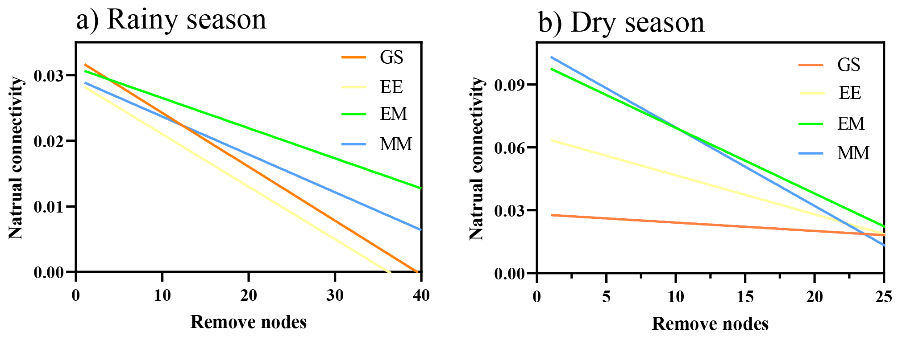


Fig. S2. Natural connectivity of soil microbial networks in relation to the number of removal nodes in rainy season (a) and dry season (b) in different management patterns.
